# The *Chop* Gene Contains an Element for the Positive Regulation of the Mitochondrial Unfolded Protein Response

**DOI:** 10.1371/journal.pone.0000835

**Published:** 2007-09-12

**Authors:** Tomohisa Horibe, Nicholas J. Hoogenraad

**Affiliations:** Department of Biochemistry, La Trobe University, Melbourne, Victoria, Australia; Baylor College of Medicine, United States of America

## Abstract

We have previously reported on the discovery of a mitochondrial specific unfolded protein response (mtUPR) in mammalian cells, in which the accumulation of unfolded protein within the mitochondrial matrix results in the transcriptional activation of nuclear genes encoding mitochondrial stress proteins such as chaperonin 60, chaperonin 10, mtDnaJ, and ClpP, but not those encoding stress proteins of the endoplasmic reticulum (ER) or the cytosol. Analysis of the chaperonin 60/10 bidirectional promoter showed that the CHOP element was required for the mtUPR and that the transcription of the *chop* gene is activated by mtUPR. In order to investigate the role of CHOP in the mtUPR, we carried out a deletion analysis of the *chop* promoter. This revealed that the transcriptional activation of the *chop* gene by mtUPR is through an AP-1 (activator protein-1) element. This site lies alongside an ERSE element through which *chop* transcription is activated in response to the ER stress response (erUPR). Thus CHOP can be induced separately in response to 2 different stress response pathways. We also discuss the potential signal pathway between mitochondria and the nucleus for the mtUPR.

## Introduction

Mitochondria serve critical functions in the maintenance of cellular energy supplies, thermoregulation, synthesis of essential molecules such as phospholipids and haem, and in apoptosis. Since mitochondrial proteins are encoded by nuclear genes (at last estimate, about 1500 [1]) as well as mitochondrial genes (encoding just 13 polypeptides in mammalian species [2]), the normal functions of mitochondria require the coordination of two genomes and a system of communication between two organelles [3]–[5]. In addition, mitochondria need to respond to changes in the physiological milieu of the cell to repair damage caused by mutations in mtDNA which produces modified proteins which are unable to fold and become prone to aggregation.

Metabolic cues and other changes which occur within mitochondria can culminate in wide-ranging changes in nuclear gene expression via retrograde mitochondrial to nuclear signaling. These responses are broadly referred to as mitochondrial stress responses [6], [7] and are generally defined as a response to altered mitochondrial membrane potential or uncoupling of oxidative phosphorylation. This leads to the elevation of cytosolic Ca^2+^ and activation of CaMK and calcineurin responsive genes [4] which include genes involved in Ca^2+^ transport and storage [6] as well as a large collection of transcription factors [8]. The net effect of activation of this gene network is to facilitate recovery of the physiological functions of the mitochondrion.

A unique type of mitochondrial stress is the mitochondrial unfolded protein response (mtUPR, which we have previously called ‘the mitochondrial stress response’ [9]) where the accumulation of unfolded proteins in the mitochondrial matrix leads to an increase in nuclear encoded mitochondrial chaperones and protease, which facilitate the recovery of function by refolding or by removal of unfolded proteins [9]–[11]. Indeed, the changes in levels of these quality control proteins in the mitochondrion exactly overlap with the changes in level of protein aggregates in the organelle [9].

We have previously shown that mtUPR responsive genes are activated through a CHOP element and transcriptional activation requires the hetero-dimerisation of the C/EBP homology protein CHOP and C/EBPβ (CAAT enhancer-binding protein) [9]. However, the gene encoding CHOP is itself activated by the mtUPR suggesting that the *chop* promoter contains a mtUPR response element. Similarly, the erUPR also results in the transcriptional activation of the *chop* gene and it has recently been shown that elevation of CHOP in erUPR culminates in the elevation of the pro-apoptotic factor BIM and apoptosis [12].

In this paper, we describe the identification of an mtUPR response element and components of a signaling pathway that leads to the transcriptional activation of the *chop* gene in response to the accumulation of unfolded protein in the mitochondrial matrix of mammalian cells. In an accompanying paper [13], we describe features of the promoters of mtUPR responsive genes that are activated by CHOP and C/EBPβ in response to the accumulation of unfolded proteins in mitochondria.

## Results

### Transcriptional activation of chop

**Figure 1:**
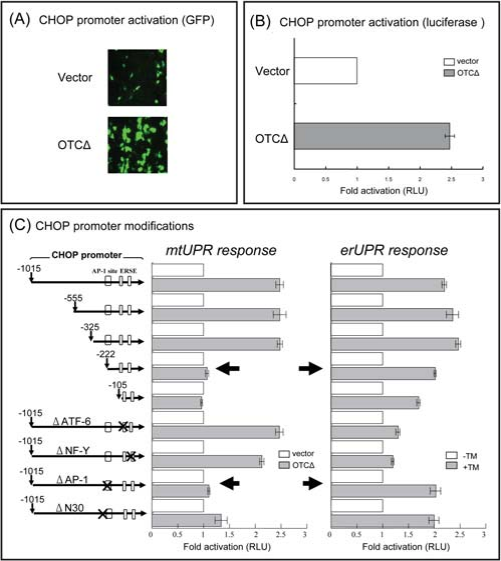
Identification of mtUPR response element in *chop* promoter. (A) and (B): *Chop* is transcriptionally activated by mtUPR. COS-7 cells co-transfected with empty vector or OTCΔ were assayed for GFP (A) or luciferase (B) 32 h after transfection. (C): identification of an mtUPR element in the *chop* promoter was determined by a deletion analysis as shown. Deletions are shown as distance (bp) from the *chop* transcription start site. The fold activation of the promoter constructs in cells transfected with OTCΔ (slash bars) are compared with vector controls (open bars) as relative luciferase (RLU) activity. RLU activity of the promoter constructs in cells treated with or without 2 µg/ml tunicamycin (TM), to produce erUPR is shown as a control. Data represents the mean±SEM from experiments performed in triplicate.

We have previously developed an experimental model for production of a mtUPR and have shown that a mutant of the mitochondrial matrix protein containing a small deletion of one of the substrate binding sites in ornithine transcarbamylase (OTCΔ) was imported into the mitochondrial matrix normally in COS-7 cells, but upon cleavage of the mitochondrial pre-sequence formed aggregates and induced genes encoding the mitochondrial chaperonins, chaperonin 60 (Cpn60) and chaperonin 10 (Cpn10) as well as the matrix protease ClpP [9]. Moreover, we showed that OTCΔ induces transcription of *cpn60* and *chop*, but not the ER isoform of Hsp70 (Bip), in COS-7 cells. Creation of an erUPR by the addition of either tunicamycin or thapsigargin to COS-7 cells in contrast, strongly induces the ER isoform of Hsp70 and CHOP, but had only a minor effect on transcriptional activation of the *cpn60* gene [9]. Thus, the accumulation of unfolded proteins leads to a specific response in each organelle, despite the fact that both UPRs induce transcription of the *chop* gene. Since Northern analysis measures steady-state concentration of mRNA, the experiments were repeated using *chop* promoter constructs. As shown in Figure 1A, expression of OTCΔ lead to an activation of a *chop-gfp* promoter construct of approximately 2.3 fold whereas a quantitative assay using luciferase as the reporter enzyme shows that OTCΔ activates transcription approximately 2.5 fold over the constitutive transcriptional activity obtained from cells transfected with vector without the OTCΔ insert (Figure 1B).

The specificity of induction of CHOP by organelle specific UPRs suggests that the *chop* promoter contains separate elements for activation in response to erUPR and mtUPR.

### Identification of an mtUPR element in the chop promoter

**Figure 2:**
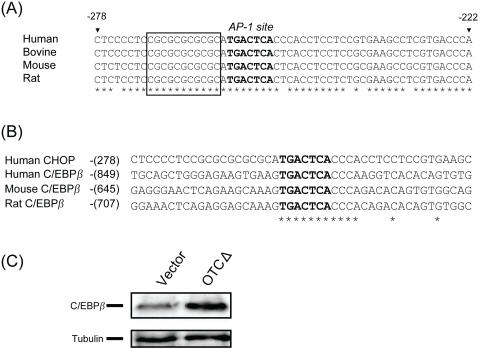
*Chop* and *c/ebp*β promoters contain AP-1 sites and are inducible by mtUPR. (A) Nucleotide sequence alignment of the mammalian *chop* promoters (−278 to −222) from human, bovine, mouse and rat. Bold letters show the highly conserved bases of the AP-1 site and the asterisks show the highly conserved sequence surrounding the AP-1 site in *chop* promoters. The position of the putative novel element of N 30 [18] is shown in the box. (B): Nucleotide sequences of mammalian *c/ebp*β promoter region around AP-1 site (Human, Mouse, and Rat) is compared with the *chop* promoter sequence (−278 to −233). The numbers refer to the distance from transcription initiation site of human *chop* or human, mouse, and Rat *c/ebp*β. The asterisks indicate the conserved nucleotides around the AP-1 site. (C): C/EBPβ expression in response to mtUPR. Extracts from cells transfected with vector or OTCΔ were subjected to western blotting and probed with antibodies against C/EBPβ and tubulin as control and show that C/EBPβ, like CHOP is induced by expression of OTCΔ.

A deletion analysis of the *chop* promoter between bases −1015 and +17 (zero being the transcriptional start site) was carried out by assaying promoter activity using the luciferase reporter enzyme. MtUPR activity was measured by comparing the activity obtained from cells transfected with OTCΔ compared with empty vector and erUPR activity was measured by adding tunicamycin to cells transfected with the promoter-LUC construct. Deletions between −1015 and −325 had no effect on *chop* transcriptional activity (Fig 1C), whereas a further deletion of 103bp essentially ablated mtUPR inducible *chop* promoter activity. With respect to erUPR inducibility of *chop*, the critical element appears to lie between −105 and +1 bp (Fig 1C and Fig 2). A sequence comparison of *chop* promoters from human, bovine, mouse, and rat shows that this region between −278 and −222 contains an AP-1 site (Figure 2A), whereas the previously identified ERSE [14], [15] lies between −105 bp and +1 bp (data not shown). The ERSE element consists of two transcription factor binding sites, ATF-6 [15] and NF-Y [16], [17]. We deleted the AP-1, ATF-6, and NF-Y sites to determine if any of these sites were required for the regulation of CHOP expression in response to mtUPR. The deletion of the AP-1 site ablated the mtUPR responsiveness (Fig 1C). In contrast, the deletion of either ATF-6 or NF-Y elements, although substantially reducing the erUPR responsiveness (Fig 1C), did not remove the mtUPR responsiveness. Conversely the deletion of the AP-1 site had no effect on the erUPR responsiveness of the *chop* promoter, although the deletion of the NF-Y site did reduce the overall activity of the *chop* promoter.

As shown in Figure 2A, the promoter region flanking the AP-1 site is highly conserved in other mammalian *chop* promoters. These flanking regions may contain additional information for the activation of the *chop* gene by mtUPR. One of these regions contains a sequence homologous to a putative element, N30, previously identified in a homology search of promoter regions in a range of animal species [18] (Fig 2A, boxed sequence). Deletion of this element had a partial effect on the mtUPR responsiveness of the *chop* promoter (Fig 1C).

Since we previously showed that CHOP induces transcription of mtUPR responsive genes in combination with C/EBPβ [9], it was of interest to note that the promoter of *c/ebp*β gene also contains an AP-1 site with highly conserved nucleotides (CCCA) in the region flanking the AP-1 site (Fig 2B). This site in the *c/ebp*β gene is also highly conserved between human, mouse, and rat promoter (Fig 2B) and therefore, we should expect both *chop* and *c/ebp*β transcription to be elevated by mtUPR. This was confirmed by Western blot analysis (Fig 2C). It has recently been shown that CHOP combines with C/EBPα or β to activate BIM transcription and apoptosis in response to erUPR [12]. However, the *c/ebp*α promoter does not contain an AP-1 site (data not shown). This raises the question whether mtUPR also induces apoptosis.

### Involvement of JNK2 in mtUPR signaling

**Figure 3:**
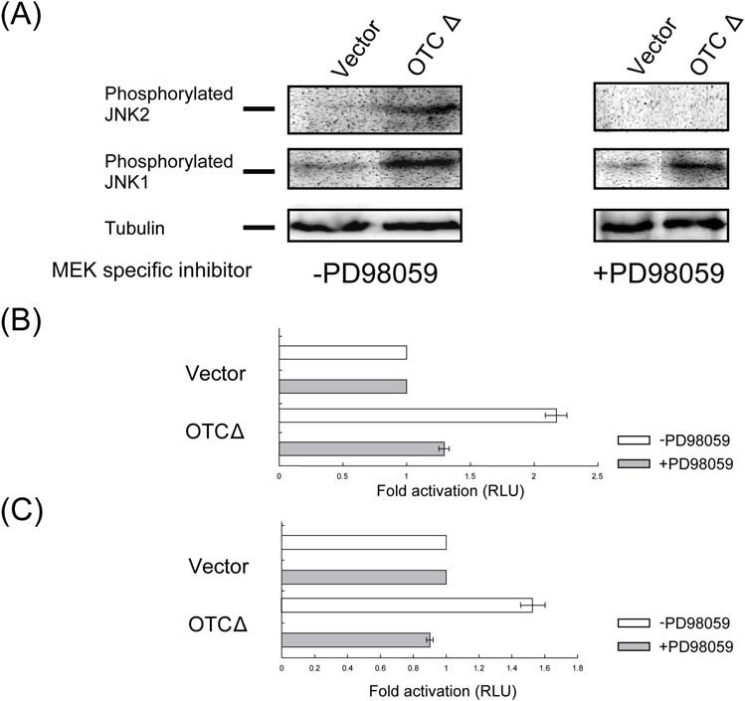
MtUPR increases phosphorylation of JNK and a MEK specific inhibitor blocks mtUPR. (A): mtUPR increases phosphorylation of JNK 1&2. Extracts from cells transfected with vector or OTCΔ, and treated with or without 10 µM of MEK specific inhibitor PD98059, were subjected to Western transfers probed with antibody against p-JNK. (B) and (C): mtUPR induction of the *yme1l1*(B) and *mpp*β(C) promoter is inhibited by the MEK specific inhibitor, PD98059. COS-7 cells co-transfected with vector or OTCΔ and *yme1l1*and *mpp*β promoter-reporter constructs, with or without 10 µM of PD98059 were used for luciferase assay 32 h after transfection. The fold activation of the promoter constructs in cells expressing OTCΔ compared with those expressing vector alone, with or without of PD98059, is shown as relative luciferase (RLU) activity. Data represent the mean±SEM from experiments performed in triplicate.

Since it is well-known that c-Jun, which is activated by JNK (c-Jun N-terminal kinase), binds to the AP-1 site [19] and it has been reported that the activation of JNK-dependent ATF2 (activated transcription factor 2) is important for the signaling from mitochondria to nucleus during the both genetic and metabolic stresses of mitochondria [3], [20], we therefore investigated the effect of mtUPR on the phosphorylation of JNK1 and JNK2 (Fig 3A). The expression of OTCΔ in COS-7 cells had a substantial effect on the phosphorylation of JNK1 and 2 (Fig 3A). To further test the potential role of JNK1 and JNK2 in mtUPR signaling, we determined the effect of the MEK inhibitor PD98059 [21], [22] on JNK phosphorylation in response to expression of OTCΔ. As shown in Figure 3A, the inhibitor completely blocked mtUPR dependent phosphorylation of JNK2, but had only a small effect on JNK1 phosphorylation. These experiments were followed up by measuring the effects of PD98059 on OTCΔ dependent activation of the mtUPR responsive promoters *yme1l1* [13] (Figure 3B) and *mpp*β [13](Fig 3C). As shown in Figure 3B and C, 10 µM MEK inhibitor inhibited the OTCΔ inducible activation of the promoter-luciferase reporter constructs in transfected COS-7 cells. This suggests that mtUPR signaling utilizes the MEK/JNK2 pathway.

## Discussion

The evolution of the eukaryotic cell facilitated the development of increased metabolic and functional complexity by dividing cells into distinct, membrane enclosed compartments. However, these organelles/compartments are extremely crowded, both in terms of small solutes and macromolecules. Thus, it has been estimated that the cytosol has a protein concentration of around 350 mg/ml [23] and the concentration inside the mitochondrial matrix may approach 500 mg/ml [24]. Not surprisingly then, the cell has evolved stress response mechanisms which come into play under conditions where unfolded proteins accumulate, such as the heat shock response [25]. Equally, the cell has evolved mechanisms to respond to the accumulation of unfolded proteins in organelle compartments such as the ER, which has become known as the UPR [14], [15], [26], [27]. This response, which was initially discovered in baker's yeast [28] has been extensively investigated and is characterized by the transcriptional regulation of a large group of genes and post transcriptional regulation of proteins involved in quality control of the secretory pathway [14], [15], [26], [27].

We discovered an equivalent stress response pathway in mitochondria of mammalian cells [9], [10] and originally called it the Mitochondrial Stress Response. More recently, Ron and colleagues discovered the response in *c.elegans* [11] and more appropriately called the response the mtUPR, distinguishing it from the erUPR, as we have done in this paper. Surprisingly, the mtUPR has not been found in fungi and appears to be an organelle specific stress response found only in multi-cellular organisms.

We originally found that in the mammalian mtUPR responsive gene *cpn60/10*, the CHOP and C/EBPβ transcription factors were involved in transcription regulation [9]. However, since mtUPR also led to the transcriptional regulation of *chop*, this suggested that the induction of the *chop* gene is an early event in mtUPR. We were also intrigued by the finding that although there appears to be little overlap in the mtUPR and erUPR, both responses led to the induction of *chop* transcription. In this paper we describe the identification of an mtUPR response element in the promoters of both *chop* and *c/ebp*β genes. This element is an AP-1 site, suggesting that mitochondrial to nuclear signaling of the accumulation of unfolded proteins in the mitochondrial matrix is through a JNK pathway. We show, using a specific MEK inhibitor, that this signaling is through JNK2 and that an inhibition in the phosphorylation of JNK2 also inhibits mtUPR. We suggest that the cell can discriminate between organelle specific unfolded protein responses through different pathways to activate genes that harbor different stress elements within their promoters. Recently, it has been reported that JNK2 is a positive regulator of the cJun transcription factor [29], and can regulate both mitochondrial and lysosomal death pathways in mouse embryonic fibroblasts [30]. This, taken together with the data presented here, suggests that the JNK2 pathway may play a significant role for the communication from mitochondria to the nucleus in response to mtUPR. Since both mtUPR and erUPR activate transcription of a distinct set of genes, yet both induce CHOP, it is apparent that additional factors besides CHOP and C/EBPβ account for the specificity of the mtUPR. This specificity is provided for the erUPR by the transcription factors ATF6 and NFY [14.15]. The question of the specificity of mtUPR is further explored in the accompanying paper [13].

Recently, Benedetti *et al.* [31] have carried out a search for genes involved in signaling of mtUPR in *c.elegans* and discovered the involvement of the *ubl-5* gene, encoding the ubiquitin-like protein 5. Whether this pathway exists in mammalian cells, or whether this pathway in *c.elegans* intersects with the pathway we describe here is currently unknown, as is the question whether the CHOP based response described in this paper operates in *c.elegans*.

## Materials and Methods

### Materials

Tunicamycin was purchased from Sigma Chemical (St Louis, USA). MEK inhibitor, PD98059, anti-C/EBPβ, and anti-pJNK were purchased from Santa Cruz Biotechnology (Santa Cruz, USA). All reagents were of reagent grade quality.

### Plasmid construction, transfection and promoter analysis

Mammalian expression vectors of wild-type OTC and deletion mutant OTCΔ were constructed as described previously [9]. Transfection efficiencies were between 72 and 85% as determined by transfections with a GFP construct. Based on the human genome sequence information of NCBI, the promoter region of CHOP (from −1015 to +17) was amplified by PCR [32] from human genomic DNA (Promega, Madison, USA) using 5′-CTTTTGGGAGAT**C**TACGGGGCTAGAACAGGAGACCACCC-3′ and 5′-GATACGCTCAG**AAG**CTTAGACTTAAGTCTCTGACCTCGG-3′ as the upper and lower primers, respectively (mutated nucleotides to introduce *Bgl*II and *Hind* III are underlined), and cloned into *Bgl*II-*Hind* III sites of the pGL3-Basic vector (Promega, Madison, USA), which contains the firefly luciferase coding sequence but lacks eukaryotic promoter or enhancer elements. For the GFP assay of promoter constructs, luciferase was replaced by GFP cDNA using *Nco* I – *Xba* I sites in the pGL3-Basic vector. Deletion mutants of the CHOP promoter were constructed by PCR using 5′-GGGGCCAAGA**G**AT**C**TGGGAGTCCCTTATAG-3′(−555), 5′-GACACCGGTTGCCA**G**A**TC**TTGCATCATCCCCGCC-3′(−325), 5′-CCGTGAAGCCTCG**A**GA**T**C**T**AAAGCCACTTCCGGG-3′(−222), and 5′-GGCGGATGCGA**A**G**AT**C**T**GGGCGGGGCCAATGCC-3′(−105) as upper primers, respectively, and 5′-GGTGGCTTTACCAACAGTACCGGAATGCC-3′ as lower primer (mutated nucleotides are underlined) for wild type CHOP promoter introduced into PGL3-Basic vector. For disruption of ATF-6, NF-Y, and AP-1 transcription factors [14], [18] or point mutation, site-directed mutagenesis was carried out by PCR [32] using 5′-GCCGGCG**G**GCCACTTTCTGATTGGTAGG-3′ and 5′-CCTACCAATCAGAAAGTGGC**C**CGCCG-3′ for ΔATF-6, 5′-GCCGGCGTGCCACTTTCTGAT**G**GGTAGG-3′ and 5′-CCTACC**C**ATCAGAAAGTGGCACGCCG-3′ for ΔNF-Y, 5′-GCGCGCGCATGA**AA**CACCCACCTCCTCCGTG-3′ and 5′-GAGGCTTCACGGAGGAGGTGGGTG**TT**TCATGCG-3′ for ΔAP-1, and 5′- CACTCCCCTCCGC**AAA**CGC**A**CATGACTCACCCACCTCCTCC-3′ and 5′- GGAGGAGGTGGGTGAGTCATG**T**GCG**TTT**GCGGAGGGGAGTG-3′ for ΔN30 [33] as the upper and lower primers, respectively (mutated nucleotides are underlined).

COS-7 cells were cultured in DME/5 % fetal calf serum and transfected at 90 % confluence using Lipofectamine 2000 (Invitrogen, California, USA). Promoter analysis using luciferase assay was carried out as described previously [9]. To induce erUPR, cells were treated with 2 µg/ml tunicamycin for 10h. Western blot analysis was carried out as described previously [9].
